# Functional outcomes of the knee and associated factors after intramedullary nailing of tibial diaphysial fractures at Addis Ababa Emergency, burn, and Trauma Hospital (AaEBT) hospital, Ethiopia

**DOI:** 10.1186/s12893-023-02155-8

**Published:** 2023-08-23

**Authors:** Eyob Ketema, Samuel Kebede, Shikur Mohammed, Tilahun Desta, Dereje Bayissa Demissie

**Affiliations:** 1 Orthopedics and Traumatology Surgery resident) school of Medicine, St. Paul’s Hospital Millennium Medical College, Addis Ababa, Ethiopia; 2 school of Public health, St. Paul’s Hospital Millennium Medical College, Addis Ababa, Ethiopia; 3 Assistant professor of Orthopedics and Traumatology Surgery, rehabilitation specialist, school of Medicine, St. Paul’s Hospital Millennium Medical College, Addis Ababa, Ethiopia; 4 school of Nursing, St Paul’s Hospital Millennium Medical College, Addis Ababa, Ethiopia

**Keywords:** Knee functional outcome, Tibial diaphyseal fracture, Intramedullary nailing, AaBET Hospital, SIGN nail

## Abstract

**Background:**

Tibial shaft fractures are the most common long bone fractures requiring treatment. High-energy trauma often causes tibia bone injuries, causing severe complications and long-term disability due to inadequate soft tissue coverage. Tibial shaft fractures can be treated using casts, external fixators, plating, or intramedullary nails. Intramural nailing leads to faster union and reduced complications like malunion and shortening. However, patients often report subjective and objective difficulties after Surgical Instrument generation network (SIGN) nail fixation, affecting knee range of motion, quality of life, and sport activities. Tibial nails and plates are associated with increased knee pain, which negatively affects functional outcomes. No study has been conducted in a poor resource setting like Ethiopia. This study aims to assess functional outcomes of the knee and associated factors after intramedullary nailing of Tibial Diaphysial Fractures at AaBET hospital in Ethiopia.

**Methods:**

A retrospective health facility based cross-sectional study was conducted on functional outcomes of the knee and associated factors after intramedullary nailing of tibial diaphysial fractures done at AaBET hospital. A medical record review form and a structured questionnaire from patient chart and SIGN nail database collected data. The study was conducted on 151 patients registered on the SIGN nail database using a simple random sampling. Knee injury and Osteoarthritis Outcome Score (KOOS) was used to assess the knee functional outcome. Descriptive statistics such as frequency and percentage were used to summarize the results and binary logistic regression was used to describe the association between variables. P value < 0.05 was considered statistically significant association.

**Results:**

The study constituted 151 patients with tibial shaft fractures; 113(74.8%) males and 38(25.2%) females with a mean age of 31.4 years, with a standard deviation of [10.5]. The prevalence of patients with good knee functional outcomes was 87(57.6%), while 64(42.4%) patients had poor knee functional outcomes. Associated factors identified include sex, age, soft tissue status, postoperative infection postoperative physiotherapy and comminuted fracture pattern.

**Conclusion and recommendation:**

: This study determined the magnitude of knee functional outcomes revealed that more than half (57.6% ) of patients had good knee functional outcomes with identified factors increseaes odds of poor knee functional outcomes were sex, age, soft tissue injuries, post operative infection, postoperative physiotherapy and comminuted fracture patterns respectively. Therefore, Policymakers and health planners should closely monitor postoperative physiotherapy treatment courses among tibial shaft fractures treated with intramedullary nailing to increases good knee functional outcomes.

## Background

The tibia is the most commonly fractured long bone in the human body, Tibial shaft fractures are defined as fractures that occur 5 cm distal to the tibial plateau and 5 cm proximal to the tibial plafond, tibial shaft is narrowest at the junction of its middle and distal third, which is the most frequent site of fracture, because of its subcutaneous nature it has poor blood supply [1]. Traumatic injuries cause significant morbidity and mortality in low-income and middle-income countries (LMICs) [2]. Road traffic accidents are responsible for most of these injuries, leading to over 1.2 million deaths and up to 50 million nonfatal injuries annually [3].

In a Prospective study done at Addis Ababa University, Tikur Anbessa Hospital the incidence of Tibial fractures was higher in the distal third 9(42.8%) followed by middle and proximal third each 6(28.5%) [4].

The incidence of tibial shaft fracture was 16.9/100,000/year. Males have the highest incidence of 21.5/100,000/year and present with the highest frequency between the ages of 10 and 20. In contrast, women have a frequency of 12.3/100,000/year and have the highest frequency between the age of 30 and 40. Most tibial shaft fractures occur during walking, indoor activity and sports. The distribution among genders shows that males present a higher frequency of fractures while participating in sports activities and walking. Women present the highest frequency of fractures while walking and during indoor activities [5].

Tibial fractures are associated with a wide range of injury mechanisms and severities. Although most fractures are closed, open fractures of the tibia are more commonly seen than in many other bones because of its subcutaneous location. The management of tibia fractures is significantly influencedby the associated soft tissue injury. Severe open fractures of the tibia are associated with high complication rates and poor long term outcomes [2].

The tibial diaphysis is the most common site of fracture in the tibia. The most common causes of low energy tibial fractures are falls from a standing height and sporting injuries, High energy tibial diaphyseal fractures are most commonly associated with vehicular trauma. One study showed that in 1988 to 1995 vehicular trauma accounted for 37.5% of tibial diaphyseal fractures with pedestrians being struck the most common mechanism [2]. With increased urbanization, about 37.5% of tibial fractures are related to motor vehicle accidents (MVA), followed by sports-related injury (30.9%). Tibia is the commonest long bone to sustain an open fracture (23%) [6]. The Surgical Implant Generation Network (SIGN) has developed technology that allows all orthopaedic surgeons to treat fracture patients with locked intramedullary nailing without needing image intensifiers, fracture tables or power reaming. Introduced in 1999, SIGN nails have been used to treat more than 100,000 patients in over 55 developing world countries. Studies have shown that patients return to function more rapidly, hospital stays are reduced, infection rates are low and clinical outcomes excellent [7].

A survey of 261 orthopaedic service providers in sub-Saharan Africa, involving 80% of respondents in low-middle-income countries (LMICs), found that initial debridement occurred most frequently in the operating room within 24 h. LMIC surgeons reported delays due to equipment availability, treatment cost, and OR availability. Additionally, LMIC surgeons used lower rates of internal fixation for high-grade and late-presenting fractures [8]. Another study done in Kilimanjaro Christian Medical Centre in Northern Tanzania reported 199 patients with sustained tibia/fibula fractures, with 78% male and 21–30 years being the most affected age group. Motor traffic accidents were the most common cause, with motorbikes being the most common. The most common site of injury was the distal-third of the tibia/fibula [9]. The commonest cause of the injuries was motorcycle accidents (25.7%) followed closely by motor vehicular accidents (25%). As study conducted in Nigeria identified that the most common associated injuries in order of frequency were: ipsilateral fibular fractures, ankle injuries, ipsilateral femoral fractures and pelvic fractures [10].

A study in Ethiopia found that 32.6% of the victims were between 15 and 29 years old, with lower extremity fractures being more common at 65.6% than upper extremity fractures at 34.7%. The most common fractured bone was the femur (23.7%), with transverse fractures being the most common (35.5%). Road traffic injuries (42.2%) and falling accidents (29.6% were the leading causes of fractures [11]. However, Knee pain, swelling and stiffness, restrictions in quality of life and limitations in sports remain common complications after operation with the insertion of an intramedullary nail after tibia shaft fracture [6]. Therefore, the magnitude of poor functional outcome of the knee following intramedullary fixation of the tibial diaphyseal fractures and factors associated with poor outcomes were not studied well in our setup.

## Method and materials

### Study setting and period

The study was conducted in the Orthopedics and Traumatology department of Addis Ababa Emergency, Burn, and Trauma Hospital (AaEBT) AaBET Hospital. AaBET Hospital is an affiliate of Saint Paul’s Hospital Millennium Medical College (SPHMMC), one of the largest tertiary hospitals in the country, located in Addis Ababa, Ethiopia. It is the first trauma hospital established in Ethiopia since 2015. It has been providing several services, including neurosurgery, orthopedics, trauma, burns, emergency care, and critical care, since its establishment. Since its establishment, It has provided several services, including neurosurgery, orthopedics, trauma, burns, emergency care, and critical care. It has a total of 145 beds for all departments, 4 OR tables, and 2 recovery rooms. There are 16 orthopedic surgeons and 5 plastic and reconstructive surgeons. The orthopedics and traumatology department provides service to 710 patients per month on average, of whom 460 are treated on an emergency basis and 127 undergo major operations on an elective basis. Plastics and reconstruction surgery and General surgery departments perform about 28 major surgeries each on a monthly basis. The study was conducted on patients with tibial diaphyseal fractures who were treated with SIGN nail fixation from January 2015 to January 2020.

#### Study design and population

A Retrospective health institution-based cross-sectional study design was employed among patients who were treated with SIGN nail for tibial diaphyseal fracture at AaBET hospital under orthopedic and traumatology department.

#### Sample size determination and sampling technique

A single population proportion sample size determination formula was used based on confidence interval approach. There was no determined proportion of tibial fracture functional outcome treated with IMN from previous similar study. Hence, p was taken to be 50%, with 95% confidence interval (Zα/2 = 1.96) and 5% margin of error (d = 0.05). In the study period, 408 patients operated with intramedullary nailing for tibial diaphyseal fracture (as counted from the SIGN database). Because sampling is from finite population size N = 408, the final sample size is calculated using the correctional formula The final sample size after 5% non-respondent rate was added was 172.

### Data collection procedure and instruments

The Knee injury and osteoarthritis outcome score (KOOS) was used to assess the functional outcome of the knee after intramedullary fixation of tibial diaphyseal fractures. The KOOS questionnaire was developed in the 1990s to assess patients’ opinions about their knee and associated problems. Data was collected using two questionnaires: study-specific and functional outcome questionnaires. The data was collected from the patients’ charts, operation log books, X rays and the SIGN nail database [12, 13]. It is widely used for research purposes in clinical trials, large-scale databases and registries. It consists of 5 subscales; Pain, other Symptoms, Activities of Daily Living (ADL), Sport and Recreation Function (Sport/Rec) and knee-related Quality of Life (QOL). The previous week is the period considered when answering the questions. Standardized answer options are given (5 Likert boxes) and each question is assigned a score from 0 to 4. A normalized score (100 indicating no symptoms and 0 indicating extreme symptoms) is calculated for each subscale. Prior to data collection pretesting of the data extraction tools were done on 5% of the study population. After the pretest, the questionnaire was checked for its clarity, completeness, reliability, consistency, and sensitivity. Then, corrections were made accordingly. The pretest results indicated that items were managed for internal correlation (Cronbach’s alpha (α) of 0.752) and instrument reliability determined (α = 0.759). Data was extracted by five trained 3rd year orthopaedic residents after two days of training, including practical sessions. Based on the hospital protocol patients’ medical records were completed based on Knee injury and Osteoarthritis Outcome Scores during follow-up visits scheduled was arranged before discharge after seven days for all patient underwent the procedures.

### Inclusion and exclusion

Patients treated for acute long bone fractures treated with SIGN nails and Knee injury and Osteoarthritis Outcome Scores were completed during the follow up appointments included in the study, and tibial diaphyseal fractures:

Tibial fractures excluding within 5 cm of the ankle and knee joint, Patients did not return for regular follow-ups during treatment, patients medical records with incomplete Knee injury and Osteoarthritis Outcome Scores, soft tissue injuries, and pre-existing knee pain and poor function prior to injury were excluded. Admission history was considered to determine the knee pain and function of the patients if they had pre-existing knee pain and poor function prior to injury were excluded from this study.

### Operational definition

Delayed fracture fixation was defined as fracture fixation after 30 days of injury [14].

Scoring instructions.

The KOOS’s five patient-relevant dimensions are scored separately: Pain (nine items); Symptoms (seven items); ADL Function (17 items); Sport and Recreation Function (five items); Quality of Life (four items). A Likert scale is used and all items have five possible answer options scored from 0 (No problems) to 4.

(Extreme problems) and each of the five scores is calculated as the sum of the items included [12, 15].

Interpretation of scores.

Scores are transformed to a 0–100 scale, with zero representing extreme knee problems and 100 representing no knee problems as common in orthopaedic scales and generic measures. Scores between 0 and 100 represent the percentage of total possible score achieved [12, 15]. The Knee injury and osteoarthritis outcome score (KOOS) of above 70 indicates good outcome while those less than 70 indicates a poor outcome(12, 15).

Partially completed physiotherapy means of total prescribed physiotherapy tratement at least completed 50% and completed means completed 100% of prescribed physiotherapy threatment.

### Data processing & analysis

The collected data was coded, entered and analyzed using SPSS Version 26 for Windows. It was checked for completeness, cleaned, processed and analyzed accordingly.Descriptive statistics used to present study participants’ characteristics and fitted a binary logistic regression model to identify factors associated with knee functional outcomes. Bivariable analysis was conducted to identify explanatory variables, and all explanatory variables with a p-value of less than 0.25 were included in the multivariable logistic regression analysis. Model fitness was checked using Hosmer and Lemeshow goodness of fit, and multicollinearity was assessed using the variance inflation factor (VIF). The level of significance was determined using adjusted odds ratio and P-value of less than 0 0.05.

## Result

### Sociodemographic characteristics of the study population

From a total of 408 patients who had sustained tibial shaft fractures and intramedullary nailing, only 172 were eligible to be included in the study, of which 21 patients did not return for follow-up during the study period, which was considered a non-response rate. The final sample size was 151 respondents, which gave a response rate of 151/172 = 88%. Of the total 151 respondents, 113 (74.8%) were males, with a mean age of 31.4 years and a standard deviation of [10.5]. See details in Table 1.

**Table 1:** Sociodemographic characteristics of the study participants, AABET hospital, 2022

Variable		Frequency	Percentage
Sex	Male	113	74.8
	Female	38	25.2
Age	17–33 yr	95	62.9
	34–50	48	31.8
	51–66	8	5.3

### Magnitude of functional outcomes of the knee among intramedullary nailing

**Fig. 1:**
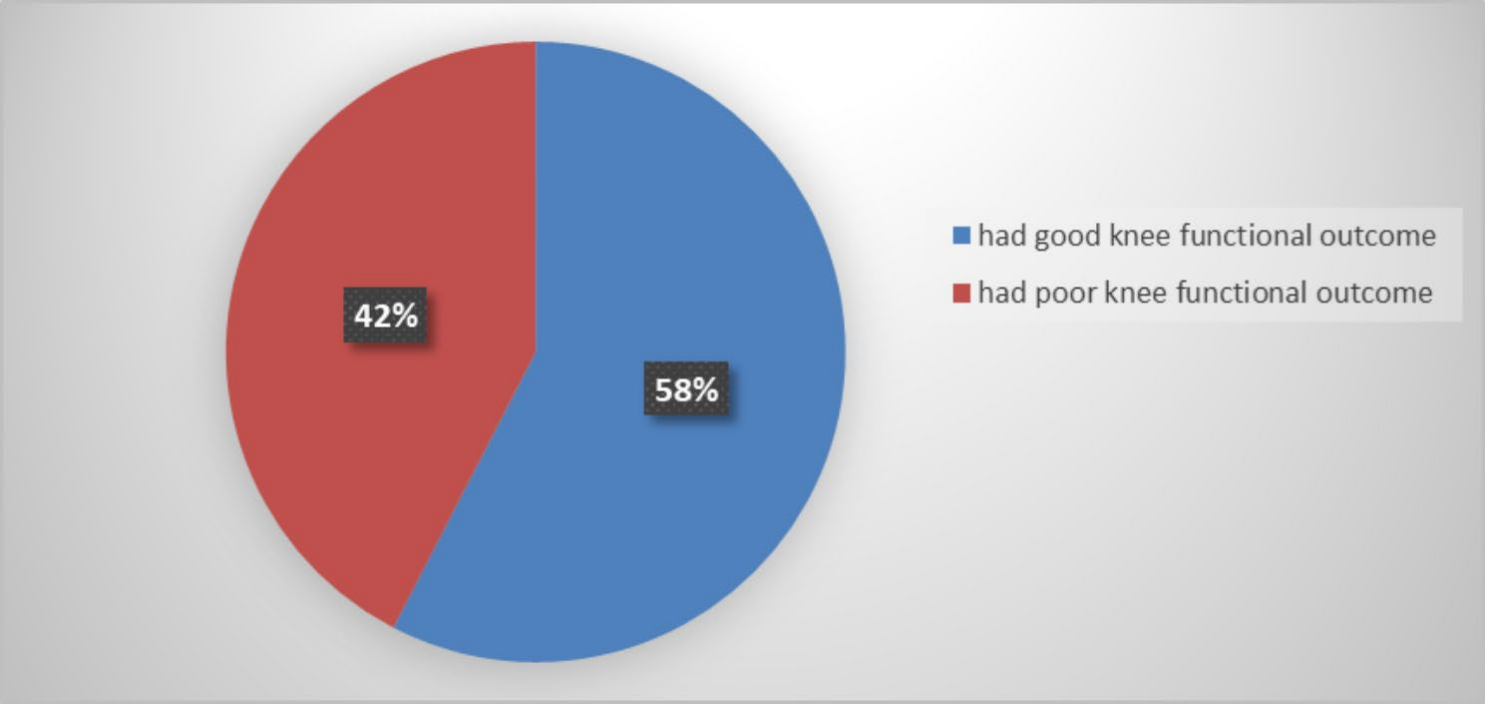
Magnitude of knee functional outcomes among Tibial Diaphysial Fractures at Addis Ababa Emergency, Burn, and Trauma Hospital (AaEBT) hospital, Ethiopia 2023

This study determined the magnitude of knee functional outcomes revealed that 87 (57.6%) of patients had good knee functional outcomes, while 64 (42.4%) patients had poor knee functional outcomes in Fig. 1.

### Variables related to the injury and past medical history of the population

The commonest mechanism of injury in this study is road traffic accident which accounts for 96(63%) cases while fall down accident accounts for 6 (4%). More than half of the patients were injured on their right side of the extremity. A majority of the sampled patients did not have associated medical comorbidity. Among those with medical comorbidity Diabetes mellitus accounted for 10(6.6%) of cases and Hypertension accounted for 10 (6.6%) of the cases. The postoperative physiotherapy treatment course was completed by 71(47%) patients while it was only partially completed in 80(53%) patients. The mean time from injury to definitive intramedullary nailing was 5.8 days with standard deviation of [5.9]. The median time was three days with a range of 1 to 28 days see details in Table 2.

**Table 2:** Distribution of history of participants related to the injury, AABET hospital, 2022

Variable		Frequency	Percentage
Mechanism of injury	Road traffic accident	96	63.0
	Fall down accident	6	7.0
	Bullet injury	7	4.6
	Other(man-made traumatic)	42	27.8
Side of the injured limb	Right	99	65.6
	Left	52	34.4
Physiotherapy treatment course	Fully completed	71	47.0
	Partially completed	80	53.0

NB: Partially completed physiotherapy means of total prescribed physiotherapy tratement at least completed 50% and completed means completed 100% of prescribed physiotherapy treatment

### Variables related to the fracture characteristics

This study had 62(41.1%) open fractures and 89(58.9%) closed fractures. Among the open fractures 26(41.9%) were Gustillo-Anderson type I, 24(38.7%) type II and 12(19.3%) were Gustillo-Anderson type III. Isolated tibial fractures occurred in 41(27.2%) patients while 110(72.8%) patients had associated Fibular fracture see details in Table 3.

**Table 3:** Distribution of participants according to fracture characteristics, AaBET hospital, 2022

Variable		Frequency	Percentage
Soft tissue status	Open	62	41.1
	Closed	89	58.9
Gustillo-Anderson classification of open fractures	GA I	26	41.9
	GA II	24	38.7
	GA III	12	19.3
Associated Fibular fracture	Yes	110	72.8
	No	41	27.2

**Fig. 2:**
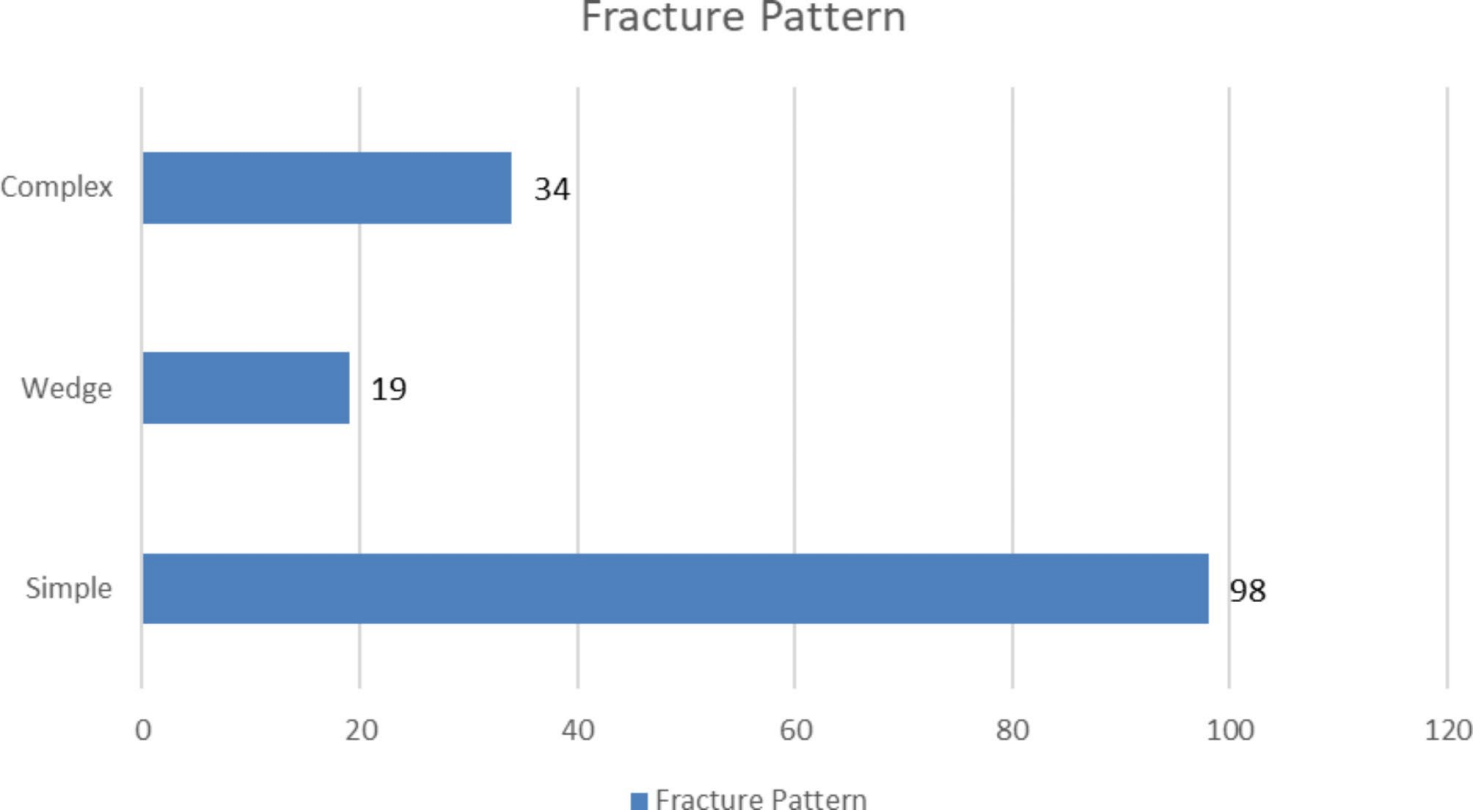
Distribution of participants according to Pattern of fracture, AABET hospital, 2022

As below Fig. 2 depicted that the commonest pattern of fracture was simple fracture,which accounts for 98(65%), followed by 34( 22.5%) were complex fractures. See details in Fig. 2.

### Multivariable logistic regression of factors associated with poor KOOS

Using the independent variables which were significant on bivariate analysis; sex, categorical age, soft tissue status, comminuted fracture pattern, presence of comorbidity, postoperative infection and postoperative physiotherapy, multivariate logistic regression was carried out by including all variables to adjust for cofounders.

After stepwise multiple logistic regression, it was found that females were 3.2 times more likely to have poor knee functional outcome scores than males [AOR 95% CI: 3.02 (1.22, 8.35].

It was also found that age groups between 34 and 50 are 3.34 times more likely to have poor knee functional outcome scores [AOR 95% CI: 3.34 (1.35, 8.27] while age groups between 51 and 63 are 23 times more likely to have poor knee functional outcome scores [AOR 95% CI: 23.6 (1.88, 297.44] as compared to age groups between 17 and 33 years.

Patients who had open fractures are 5.9 times more likely to have poor knee functional outcome score [AOR 95% CI: 5.95 (2.50, 14.1] while patients with comminuted fracture patterns were 2.8 times more likely to have poor knee functional outcome score [AOR 95% CI: 2.08 (1.01, 7.79]. Finally, this study also found that compared to patients who completed postoperative physiotherapy treatement course, patients who partially completed post operative physiotherapy treatment are 3.1 times more likely to have poor knee functional outcome scores [AOR 95% CI: 3.11 (1.37, 7.06].

However, some independent variables found to be statistically significant on bivariate analysis; including the presence of comorbidities and postoperative infection, were not significantly associated with poor knee functional outcome score after stepwise multivariate logistic regression (see details Table 4).

**Table 4:** Multivariate analysis of factors associated with poor KOOS, AaBET hospital, 2022

Variables	Knee Outcome score				
	Poor Outcome	Good Outcome	COR **(95% CI)**	AOR **(95% CI)**	P value
**Sex**					
Male	39	74	1.00	1.00	
Female	25	13	**2.56(1.04,6.27)**	**3.20(1.22,8.35)**	0.017
**Categorical Age**					
Age 17–33	29	66	1.00		
Age 34–50	28	20	**3.18(1.54,6.55)**	**3.34(1.35,8.27)**	0.009
Age 51–66	7	1	**15.9 (1.8,135.4)**	**23.6(1.88,297.44)**	0.014
**Comminuted fracture**					
Yes	20	14	**2.37 (1.08,5.16)**	**2.80(1.01,7.79)**	0.047
No	44	73	1.00	1.00	
**Postoperative physiotherapy**					
Fully Completed	21	50	1.00	1.00	
Partially completed	43	37	**2.76(1.41,5.42)**	**3.11(1.37,7.06)**	0.007
**Soft Tissue Status**					
Open fracture	37	25	**3.39(1.72,6.70)**	**5.95(2.50,14.1)**	0.000
Closed fracture	27	62	1.00	1.00	

## Discussion

This study investigated the magnitude of poor knee functional outcome scores in patients with tibial shaft fractures treated with intramedullary nailing at AaBET Hospital. The magnitude of poor knee functional outcome score among the study participants was42.4%. This finding is consistent with a previous study done which stated that half of the patients experienced functional limitation of the knee related to the fracture [16]. Another study done reported that also found that a third of patients with tibial fractures treated with IMN did not turn to previous level of recreation, this suggests that poor knee functional outcome score is a significant complication following treatment of tibial shaft fractures with IMN [17].

A study among similar population [18] found that males had higher incidence of tibial shaft fractures and the highest incidence occurred in the 10–20 age groups. This finding is consistent with this study where males accounted for 74.8% of the study participants with the highest incidence was in ages between 17 and 33 years. In this study, closed tibial shaft fractures accounted for 59% of the cases; a consistent finding was mentioned in previous done where most were closed fractures [2]. The current study found that open fractures are 5.9 times more likely to have poor knee functional outcomes, which is also consistent with the previously mentioned article [2]. In a previous study done [18], AO type A pattern of fracture was the most common of all the fracture types followed by type B and C, representing 34% of all fractures in the study, as compared to this study were AO type A pattern of fracture was the commonest, accounting for 64.9%. this differences may be due to study set up and socioeconomic status difference. In addition, Type C fracture pattern were associated with poor knee functional outcome score. A previos study reported a high number of fractures due to road traffic accidents [1]. Another study done in India also reported that road traffic accidents accounted for 37.5% of the cases of tibial fractures [6]. This figure is lower as compared to the findings in study where road traffic accidents accounted for 63% of the mechanism of injury. This may be explained by the higher incidence of road traffic accidents in this study setting. These finding showed that road triffic accidents is the global public health importnats in causing prematured deaths and injuries among productive age groups in developing coureis. In previous study done reported that age group between 18 and 34 years reported the poor KOOS scores compared to older age groups [18]. In contrast to our study were older age groups are associated with poor knee functional outcome scores. In this study, females are2.5 times more likely to have poor knee functional outcome scores; a finding which is similar to a study done,where women reported a statistically significantly poor knee functional outcome have shown a statistically significant deficit in quadriceps strength for patients after intramedullary nailing, suggesting that specific rehabilitation involving knee and thigh muscles may improve outcome for some patients [5, 6]. In current study patients who have partially completed postoperative physiotherapy treatment course are 8 times more likely to develop poor knee functional outcome scores.

### Limitation of the study

The study was a cross sectional study and hence could not establish a cause- effect relationship Retrospective clinical study. The findings can not be generalized as the population was only drawn from one hospital. The sample size of the study was small.

## Conclusion

This study determined the magnitude of knee functional outcomes revealed that more than half (57.6% ) of patients had good knee functional outcomes with identified factors increseaes odds of poor knee functional outcomes were sex, age, soft tissue injuries, post operative infection, postoperative physiotherapy and comminuted fracture patterns respectively. This study reported that the commonest mechanism of injury in this study was a road traffic accident, which accounts for 63% of cases in the study area. As vehicular trauma (RTA) are most commonly associated with tibial diaphyseal fractures, nationwide awareness creation and preventive measures should be taken.

Policymakers and health planners should closely monitor postoperative physiotherapy treatment courses among tibial shaft fractures treated with intramedullary nailing to increases good knee functional outcomes.

High suspicion and adequate treatment plans should be provided for patients with postoperative infections.

Health care providers should closely follow-up and provide appropriate management for patients with medical comorbidities.

Researchers should conduct national studies to identify and address factors affecting knee functional outcome after tibial nailing.

## Data Availability

Datasets used in the current study are available from the corresponding author upon reasonable request.

## Abbreviation and Acronyms

SPHMMC: Saint Paul’s Hospital Millennium Medical College
AaBET: Addis Ababa Burn, Emergency and Trauma center
Dr: Doctor
G.C: Gregorian Calendar
MD: Medical doctor
RTA: Road traffic accident
SIGN: Surgical Implant Generation Network
CI: Confidence Interval
KOOS: Knee injury and Osteoarthritis Outcome Score
SPSS: Statistical Package for Social Science
ETB: Ethiopian Birr
